# Evaluation of Alternative Protein and Lipid Sources for Rainbow Trout (*Oncorhynchus mykiss*): Growth, Fillet Quality, and Economic Outcomes of a Farm-Based Diet

**DOI:** 10.3390/ani16081188

**Published:** 2026-04-14

**Authors:** Alessandra Roncarati, Livio Galosi, Davide Dell’Unto, Maria Paola Francesca Bottoni, Martina Quagliardi, Emanuele Antenucci, Nicolaia Iaffaldano, Raffaele Cortignani, Pier Paolo Danieli

**Affiliations:** 1 School of Biosciences and Veterinary Medicine, University of Camerino, 62024 Matelica, Italy; alessandra.roncarati@unicam.it (A.R.); mariapaolafrancesca.bottoni@unicam.it (M.P.F.B.); martina.quagliardi@unicam.it (M.Q.); 2 Department of Agriculture and Forest Sciences, University of Tuscia, 01100 Viterbo, Italy; cortignani@unitus.it (R.C.); danieli@unitus.it (P.P.D.); 3 Department of Agricultural, Environmental and Food Sciences, University of Molise, 86100 Campobasso, Italy; emanuele.antenucci@unimol.it (E.A.); nicolaia@unimol.it (N.I.)

**Keywords:** sustainable fish farming, fish by-products, algal oil, economic analysis, flesh quality, *Oncorhynchus mykiss*, circular economy

## Abstract

Finding a sustainable approach to rainbow trout aquaculture is very important. This study aimed to evaluate the effects of an experimental diet (D2) including artisanal fishery discards (whiting fish—*Merlangius merlangus*; tub gurnard—*Chelidonichthys lucerne*; horse mackerel—*Trachurus trachurus*) and feedstuffs from the fish farmer’s farmhouse (wheat and dehulled peas) in comparison to a control diet (D1) on the growth performance and fillet quality of rainbow trout—*Oncorhynchus mykiss* (initial mean body weight: 48 ± 3 g). In D2, fish oil was substituted with algal oil. The trial was performed in flow-through basins. An economic assessment was performed, considering the most important economic indicators, based on the cost of feed and the cost to obtain fish biomass, comparing D2 to D1. A final reduction in total ammonia nitrogen in D2 water (TAN; 0.28 ± 0.01 mg/L vs. 0.42 ± 0.03 mg/L for D2 and D1, respectively) was observed. No significant differences in growth performance were observed, although there was a slight difference in the Feed Conversion Rate. Use of algal oil as a dietary ingredient ensured high-quality omega-3 fatty acids in the fish fillets, with a significant improvement in the DHA content of D2 fish (1131.0 ± 1.8 mg/100 g) compared to their D1 counterparts (435.0 ± 0.5 mg/100 g). The economic analysis corroborates the benefit of using marine fish by-products as alternative protein sources for rainbow trout aquaculture, as the D2 feed has a lower formulation unitary cost (−15.4%) than the D1 feed, suggesting that in addition to their efficacy in growing fish, marine fish by-products are a valuable protein source from an economic standpoint. Since this experiment was implemented at the farm level, these outcomes suggest the diet can be realistically and sustainably applied in the European rainbow trout farming sector.

## Simple Summary

To achieve sustainable aquaculture, it is necessary to produce feed, including feedstuffs as good sources of protein and lipids, without compromising environmental resources. In this study, a new feeding strategy, using by-products from fish processing and farm-produced vegetable ingredients, was compared to a standard diet based mainly on fish meal, soybean meal, and fish oil. Algal oil was also included in the experimental diet to assure high-quality fillets in terms of fatty acid profile. Farming and management practices were followed in a rainbow trout production cycle, and fish growth performance and fillet quality were evaluated, at the end performing an economic analysis.

## 1. Introduction

In the context of growing concern for the fate of the planet, the FAO [1] has emphasized the importance of aquatic food systems (fish and fish by-products) as drivers of employment, economic growth, social development, and environmental recovery [2]. For livestock systems to remain sustainable in the medium to long term, it is crucial to identify alternative sources of animal protein [3], especially the ones derived from fish by-products, since they offer a promising alternative to conventional sources, particularly within circular economy systems.

In recent years, many reviews have examined the potential use of fish by-products as a resource to take advantage of the different types of raw material they contain [4,5,6,7]. The term “fish by-products” is generic, indicating the remains of fish, obtained from fisheries or aquaculture, after processing for evisceration, beheading, and filleting; in contrast, trimmings are the rendered portions of the fillet when the frames are removed and these remains comprise viscera, blood, fins, tails, scales, skin, heads, and bones that can be treated to extract a variety of bioactive compounds [8,9]. In the same context, fish protein hydrolysates, which consist of short-chain peptides, can be obtained through enzymatic hydrolysis, having bioactive properties and a desirable nutritional profile, and being easily incorporated into food and nutraceutical products [10]. The use of fish by-products as feed for widely farmed fish species can be considered an ‘end of waste’ practice [11], as it facilitates the recovery and utilization of waste products, as secondary raw materials, or reusable substances in production processes. Many studies in the fishery industry have demonstrated that fish and seafood, specifically fish meal and fish oil with trimming meal and fish protein hydrolysates, can contribute to sustainable healthy diets [12,13,14,15].

In the context of fish farming, from a circular economy perspective, legislation plays a very important role, as it enables fisheries to appropriately manage fish discards and optimize the use of by-products: the reduction in waste and environmental impact was identified as a priority in the European Union regulatory framework when Directive 2008/98/EC was published, defining key concepts such as waste, recovery, and disponing and establishing the essential requirements for waste itself [16].

In central Italy, along the Adriatic and Tyrrhenian coasts, various initiatives have been undertaken to support artisanal fisheries, such as events organized by the Italian Fisheries Local Action Groups (FLAG) and several stakeholders [17,18,19]; in addition, studies on designating specific areas of harbours for the collection and processing of all marine by-products have been carried out [20].

The novel aquaculture management approach adopted along the Adriatic and Tyrrhenian coasts has the potential to support the aquaculture industry in its blue transition [21]; indeed, utilizing raw materials produced within a fish farmer’s agricultural enterprise widens the range of available alternatives to fish and fish oil, emphasizing the importance of assessing feed efficiency and cost-effective benefits [22].

In conclusion, this study was conducted to evaluate the effects of a feed incorporating raw marine protein sources, obtained from an artisanal fishery operating in the central Adriatic Sea, vegetable protein sources obtained from the fish farmer’s farmhouse and algal oil. The feed’s effects on growth performance and the fillet composition of rainbow trout reared in flow-through basins were evaluated and in addition an economic assessment of the feed was performed, considering the most important economic indicators based on the cost of feed and the cost to obtain fish biomass, in comparison to the conventional feed employed by the fish farmer.

## 2. Materials and Methods

### 2.1. Fishery Discards and By-Products

Parts of fish, including heads and tails, obtained by filleting carcasses of whiting fish (*Merlangius merlangius*) (46% dry matter; 54.3% crude protein; 8.7% crude lipids; 21.7% ash), tub gurnard (*Chelidonichthys lucerna*) (42% dry matter; 54.8% crude protein; 19% crude lipids; 23.8% ash), and horse mackerel (*Trachurus trachurus*) (63% dry matter; 46% crude protein; 28% crude lipids; 22% ash), caught from artisanal fisheries, were recovered by fishermen landing at the harbour of San Benedetto del Tronto (AP, Italy) with the aim of producing fish by-products suitable for use as a protein source in rainbow trout feed.

The discards were transported in refrigerated containers to a dedicated laboratory for processing fish remains belonging to the fish company in Sefro (MC, Italy), in which the study was carried out. The fish biomass was homogenized in grinders, stored under controlled temperature conditions, and submitted to the different processes required to obtain fish trimming meal (FTM) and fish protein hydrolysates (FPH), according to standardized procedures [5]. Microbiological risk assessment was performed to prevent biological hazards, as required by law, and no risk was identified.

### 2.2. Fish Diet Formulation and Manufacture

Two different diets, D1 (Control) and D2 (Experimental), were used (Table 1).

The diets were formulated to contain approximately 44% crude protein and 21% crude lipids. D1 was used as the Control, since it was manufactured according to standards for commercial rainbow trout feeds, which are widely available and used across Italy; specifically, it included fish meal, poultry meal, and fish oil. In D2, the fish meal and poultry meal were replaced with FTM and FPH and the fish oil was fully replaced with market-available algal oil (Veramaris, Delft, The Netherlands) and characterized by high eicosopentaenoic acid (EPA) (16% total FA) and docosahexaenoic acid (DHA) (39% total FA) contents. Including algal oil facilitated a reduction in the amount of soybean oil in the D2 formulation (Fatty acid profiles of the lipid sources are provided in the Supplementary Materials Table S1). The dietary fraction of vegetable feedstuffs (wheat and pea) comprised crops cultivated in a farmhouse owned by the fish farmer’s company and its portion was increased in D2 with the aim of reducing the amount of soy meal, thereby increasing the company’s self-sufficiency. The remaining D2 ingredients (Table 1) were adjusted to obtain a nutritional profile comparable to that of D1, while controlling for the unitary costs of the feedstuffs. Both the D1 and D2 feeds were pelletized to 4.5 mm and manufactured by the feed mill of the fish company (Cassolnovo, Pavia, Italy), using a twin-screw extruder (100 rpm, 110 °C, 50 atm). After coating, the feeds were stocked in buckets and kept in an aerated room and samples of each diet were taken for proximate composition analysis.

### 2.3. Fish and Growth Performance

A total of 600 specimens of rainbow trout (*Oncorhynchus mykiss*, initial m.b.w.: 48 ± 3 g; initial mean body length: 17 ± 1 cm) were housed in 6 tanks (6 × 1 × 0.5 m), with a 3 m^3^ volume each. There were 3 replicates for each diet (D1 and D2), and the fish were maintained at the same stocking density in all basins (n. 100/tank; 33.3 specimens/m^3^) in a flow-through system (10 L/min). The inlet waters of the trout farm were monitored continuously, with a digital monitoring multiprobe used to check the quality of water supplied to the fish basins.

The experiment lasted 89 days, and fish were fed twice a day (8 a.m. and 3 p.m.) until satiation. The fish were weighed in a bucket full of water from the rearing tank using an electronic scale (model WLC 20/A2, ±0.1 g, RADWAG, Radom, Poland), and their length was recorded with an ichthyometer (Scubla Srl, Remanzacco, UD, Italy).

In both dietary groups, main zootechnical parameters were assessed, such as the Specific Growth Rate (SGR) [23]:

(1) $$SGR\;(\%/day)=100\times \frac{Ln\left(final\;weight\right)-Ln(initial\;weitht)}{trial\;duration\;}$$

Feed Conversion Rate [23]:

(2) $$FCR=\frac{live\;weight\;gain\;(g)}{feed\;administered\;(g)}$$

Survival Rate [23]:

(3) $$SR\;\left(\%\right)=100\times \frac{final\;n.\;of\;fishes}{initial\;n.\;of\;fishes}$$

Condition Index [24]:

(4) $$CI=\;100\times \frac{fish\;weight\;(g)}{{fish\;lenght}^{3\;}\left({cm}^{3}\right)}$$

### 2.4. Water Quality

Throughout the experiment, in all tanks, the main water physico-chemical parameters (water temperature, dissolved oxygen, pH, nitrogen compounds) were monitored in situ using portable electronic devices (YSI mod. 55 and 60, Yellow Springs, OH, USA). In the laboratory, at the time of fish sampling, total ammonia nitrogen (TAN), nitrites (NO_2_-N), and nitrates (NO_3_-N) were analyzed monthly, following APHA standard methods [25], using a DR 2000 portable spectrophotometer (HACH, Lange Gmbh, Düsseldorf, Germany).

### 2.5. Quality Traits of Fish Fillet

The fish feeds were analyzed to determine their proximate compositions, according to the Association of Official Analytical Chemists procedure for moisture, protein, and ash [26]. At the end of the study, the fish were slaughtered by electrical stunning in the authorized slaughterhouse belonging to the fish company. A portion of about 50 g of skinless dorsal left muscle was collected from six fish randomly selected from each diet group; then, these samples were homogenized and subjected to proximate composition analyses (moisture, protein, lipid, and ash content) (see method used for the feedstuffs).

The moisture content was determined in duplicate according to the procedure described by the Association of Official Analytical Chemists [26]. The protein content was determined using the standard Kjeldahl copper catalyst method and the ash content using the procedure described by the AOAC [26].

Total lipids were measured using a modified version of the chloroform–methanol procedure described by Folch et al. [27]. After determining the total lipid content, fatty acids were converted to methyl esters following the method described by Christopherson and Glass [28]. Fatty acids were separated using a Carlo Erba HRGC 5160 gas chromatograph (Carlo Erba Strumentazione, Rodano, MI, Italy) with a WP-4 Shimadzu integration system (Shimadzu Corporation, Tokyo, Japan) equipped with a Supelco SPTM-2340 capillary column (30 m × 0.32 mm i.d.; 0.20 μm film thickness; Supelco, Bellefonte, PA, USA) and a flame ionization detector. The operating conditions of the gas chromatograph were as follows: the oven temperature was set at 170 °C for 15 min and subsequently increased to 190 °C at a rate of 1 °C/min, increased to 220 °C at a rate of 5 °C/min, and held at this temperature for 17 min. The temperature of the injector was 270 °C, while the temperature of the detector was 300 °C. Helium was used as the carrier gas at a constant flow of 1.7 mL min^−1^. The concentration of a single fatty acid was calculated based on the relative proportion of its peak area compared with the peak area of the known amount of the internal standard (17:0) added. The fatty acid content was expressed as g/100 g of fillet.

### 2.6. Statistical Analyses

The data collected (final productive traits, fish fillet proximate composition, and fatty acid categories) were subjected to Student’s *t*-test using GraphPad^®^ (version 10.4.0-621, Dotamatics, Boston, MA, USA) to check for differences in the productive performance and fillet composition of the fish fed the two diets. Means and standard deviations were calculated. Significance was set at a *p* value < 0.05.

### 2.7. Economic Analysis

The indicators used in the economic analysis refer to two types of costs: (i) the cost of feed (CF) and (ii) the cost to obtain one kilogram of fish biomass (CFB). For the first indicator, the data used are the prices and composition (parts per thousand) (Table 1) of the feedstuffs used in the trial. Using these two types of data, it was possible to calculate the weighted average that provides the CF (EUR/kg feed). Subsequently, using the Feed Conversion Rate value, the CFB (EUR/kg fish) was calculated by multiplying the two factors.

## 3. Results

Water quality, temperature (13.1 ± 0.5 °C), dissolved oxygen levels (8.3 ± 0.4 mg/L), and pH (7.8 ± 0.5) were constant in all tanks for both groups throughout the experiment. The water chemistry showed a very similar trend in terms of nitrogen compounds at different sampling times (Table 2), excepted for TAN in the D2 tanks, which was significantly lower than in the D1 tanks at the end of the experiment.

**Figure 1:**
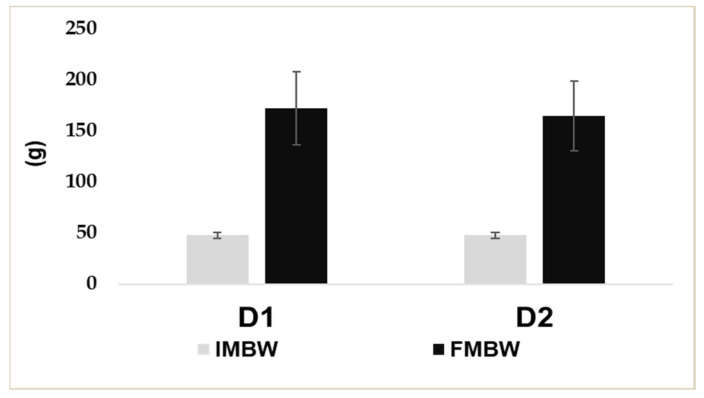
Initial (IMBW) and final mean body weight (FMBW) (±SD) of rainbow trout fed D1 (Control) or D2 (experimental) diet.

No significant differences in final mean body weight were recorded between D1 (172 ± 38 g) and D2 (164 ± 35 g) fish (Figure 1). The same trend was observed for the other productive parameters recorded and reported in Table 3. The most favourable FCR results were obtained in the D2 group.

As for the growth performance results (Table 3), no differences were recorded except for the FCR, which was about 8% lower (*p* < 0.05) in the D2 fish compared to the Control (D1) group.

**Figure 2:**
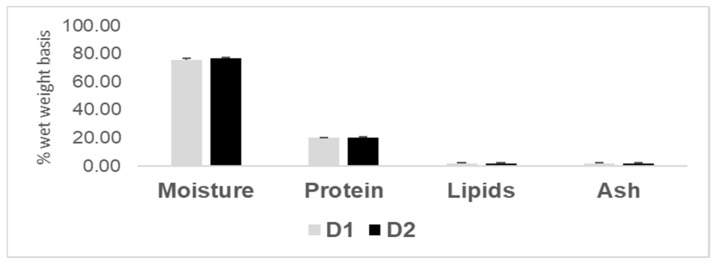
Proximate composition of trout fillets obtained from fish fed D1 or D2 diet.

No significant difference was found in the fillet macronutrient composition (Figure 2). In both groups, the protein fraction amounted to about 20%, expressed on a wet weight basis, whereas the lipid fraction ranged between 1.7% and 2.0%. The ash content remained at about 1.5% and 1.0% for the D1 and D2 fillets, respectively.

**Figure 3:**
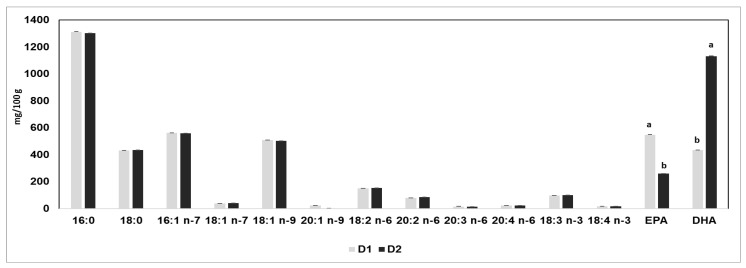
Fatty acid profile (mean ± SD) of the fillets obtained from fish fed D1 (Control) or D2 (experimental) diet. ^a,b^ *p* < 0.05 between groups within the same fatty acid.

Regarding the fatty acid profile of the fillets (Figure 3), significant differences were recorded in n-3 PUFAs between the two groups. In particular, the content of EPA was about double in D1 (548 ± 0.6 mg/100 g) with respect to D2 (258 ± 0.8 mg/100 g) (*p* < 0.05), whereas the DHA content was higher in D2 (1131 ± 1.8 mg/100 g) and lower in D1 (435 ± 0.5 mg/100 g) (*p* < 0.05).

The main results obtained from the economic analysis concerning CF and CFB are reported in Table 4.

The economic analysis showed that the D2 feed was more economically sustainable than D1, both in terms of feed cost and in terms of fish biomass production cost.

## 4. Discussion

In this study, the by-products of fish processing were used in an experimental feed to replace fish meal and fish oil, which are conventional raw materials characterized by high costs associated with their environmental impact due to the risk of overexploitation of marine stocks. Although this type of study has been performed before, this work aimed to bring together stakeholders of the Italian middle Adriatic Sea, combining the needs of a trout farmer seeking to improve the sustainability of their practices and reduce the cost of producing rainbow trout with the needs of local fishermen practicing artisanal fishery, who want to reduce the discards produced by processing harvested fish. This study demonstrates that growth performance and economic sustainability can be achieved without compromising fillet quality, addressing the three pillars of sustainability (environmental, social, and economic) [29].

To meet amino acid and protein requirements, different ingredients of animal and vegetable origin were used. The feedstuffs of animal origin were all by-products of animal processing, such as fish trimming meal, that provided the highest fraction of the protein source (32%) and were obtained from fish processed by artisanal fisheries in the Adriatic area. This ingredient was followed by hemoglobin (13.7%), which was obtained by processing swine blood via spray-drying, and subsequently hydrolyzed protein (1.85%), which was provided by fish peptides and amino acids after hydrolysis. Poultry meal was used in D2 at a lower rate than in D1 (8.53% vs. 11.4% of the protein source), which included this by-product since it is still considered a valuable alternative to fish meal [30,31,32,33]. However, in D2, poultry meal was further reduced in favour of fish trimming meal, with the aim of reducing the feed production cost.

The mix of by-products of aquatic and terrestrial animal processing proved to be essential to ensuring an amino acid profile characterized by appropriate amounts of the essential amino acids for optimal fish growth, particularly lysine and valine. The experimental feed included another sustainable source of raw ingredients, such as plant-based feedstuffs, which satisfy the EU’s most recent requirements for environmental sustainability; these raw materials were obtained from the owner’s farmhouse, which had over 2000 hectares of cultivated land. The on-farm availability of wheat and peas allowed for the inclusion of these protein-rich ingredients without the need to purchase them from the market; in particular, the availability of dehulled peas guaranteed the removal of tannins, which are associated with hulls and known to limit growth performance [34]. This condition presents a strategic option for promoting the use of plant-based feed in aquaculture, highlighting how this study’s approach ensured that the raw materials used were sourced from the local area, thereby improving the perception of sustainable production systems by large-scale retail trade and other stakeholders [35].

Favourable water quality results were obtained, as all the physico-chemical traits monitored maintained good values, especially TAN at the end of the trial period, demonstrating that the experimental diet was well-formulated, avoiding excess ammonia release. This condition is essential to prevent dispersion of toxic substances and/or nutrients in the water environment and agrees with previous studies on the importance of aquafeed processing techniques to environmental preservation [36]. The results of this study demonstrate that aquaculture is not merely an economic activity, but an essential aspect of broader objectives related to sustainable resource management and regional development, all of which align with the United Nations Sustainable Development Goal 14 (SDG 14) [37].

This work revealed some very interesting differences in the fatty acid profiles of the D1 and D2 fish fillets (Figure 3). In fact, though similar values were observed for all saturated, monounsaturated, and n-6 polyunsaturated fatty acids between the two experimental diets, the D1 trout fillets were richer in EPA than their D2 counterparts, aligning with the different concentrations of this n-3 PUFA found in the lipid sources used to formulate diets (Table S1). In rainbow trout, the metabolism of fatty acids, especially PUFAs, is limited, so the conversion of EPA to DHA is of minor importance in comparison to differential dietary intakes [38]. In contrast, a very high level of DHA was found in the D2 fish fillets compared to the D1 fillets and, as the fillet FA contents reflect the dietary contents, it is reasonable to assume that the substitution of fish oil and, although only partially (−10.5%), of soybean oil with high-DHA (39% of FA) algae oil resulted in an increased DHA content in the fillets of fish fed the D2.

Hossain et al. [39] included a high-DHA commercial algae meal in fish feed to ensure a DHA content of about 1.8 g/100 g [39], reporting a DHA level of 9.6% FA in sobaity sea bream (*Sparidentex hasta*) fillets (i.e., 560 mg DHA/100 g, 5.76% average lipid content), which is in agreement with the results of this study when considering that the DHA in the D2 feed was approximately twice as high (i.e., 3.4 g/100 g feed), primarily due to the inclusion of algal oil. However, the contribution of fish trimming meal to the increased DHA level observed in D2 fillets cannot be disregarded as, for example, in the heads of jack mackerel (*T. murphj*), where a high level of DHA (11.7 ± 0.5% FA) was recorded in comparison to EPA (3.3 ± 0.5%FA) [40]. DHA/EPA *ratios* from 3.1 to 4.5 have been reported for tub gurnard (*Trigla* sp.) and for whiting fish by Özogul et al. (2007) [41], suggesting that a prevalence of DHA over EPA might also be expected in their by-products, which were used as alternatives to FM in the D2 feed. Commercially available algal oil was incorporated into the diet to guarantee excellent fillet quality by providing an adequate source of omega-3 fatty acids; this could be of considerable importance for consumers’ health and well-being [42]. In this way, the recovery of protein sources from the farm, improving sustainability, is combined with nutritional benefits, represented by the use of algal oil as a source of -long chain polyunsaturated fatty acids, particularly EPA and DHA. This combination is relevant in the context of the recent debate on the importance of including sustainable aquaculture products in healthy human diets [43,44]. The productive performance exhibited by the fish fed the experimental diet was not different from that of the control fish, although the final mean body weight in both groups was slightly lower than values reported in other studies using poultry by-products [45] or algal oil [46]. However, it is important to consider that the current study was performed under farming conditions at the end of the most intensive reproductive season (May) to avoid interfering with routine management activities. In addition, retaining some soybean oil and not fully replacing it with algal oil as a lipid source limited the cost of the raw materials included in the experimental feed.

Concerning the economic analysis, the indicators calculated allowed for a comparison of outcomes associated with the D1 and D2 diets, considering the unit cost of the feed (CF) and the cost of producing one kilogram of fish biomass (CFB); these indicators are similar to those employed in the literature to conduct comparative assessments of alternative diet formulations for rainbow trout in aquaculture. For instance, Tefal et al. [47] compared the Price of the Diet (corresponding to the CF in the present study) for organic formulations based on alternative protein sources in place of fishmeal, evidencing the economic convenience of a mix of seabass and pig by-products to achieve the lowest price, despite the poorer growth rate compared to that achieved with fishmeal. Vergara-Rubín et al. [48] computed an Economic Conversion Ratio (very similar to the CFB) to evaluate the economic efficiency of substituting fishmeal with poultry by-product meal, highlighting its convenience only during periods of rapid growth and not across the entire production cycle. The same indicator was considered by Acar et al. [49] in evaluating the substitution of fishmeal with an insect-based feed at increasing rates, evidencing that a 25% to 50% substitution achieves the best outcome from both productive and economic points of view. Again, Fanizza et al. [45] computed indicators identical to the CF and CFB to evaluate low-fishmeal diets formulated with alternative protein sources, highlighting no significant impact on fish growth rate among the experimental diets and a lowest production cost achieved by partially replacing fishmeal with hydrolyzed feather and rapeseed. Our results align with the findings of the latter study: with comparable weight gain and quality traits, the experimental diet showed a 15.3% lower CF; as an effect of the significantly lower Feed Conversion Rate, the CFB is 21.8% lower, indicating the overall economic benefit of the D2 diet.

## 5. Conclusions

In this study, an experimental aquafeed incorporating algal oil and raw materials from a local fish farmer’s farmhouse and local artisanal fishery was investigated to evaluate its economic convenience and the growth performance and fillet quality of fish raised on the diet. The results show that the experimental diet was successful and did not compromise either rainbow trout growth or the fillet quality in terms of n-3 PUFAs. In addition, the economic indicators show that the experimental feed improved the conversion rate compared to the conventional feed used by the fish farmer.

## Figures and Tables

**Table 1:** Proximate composition, energy, and formulation (g/kg) of the two experimental diets.

Item	D1	D2
Moisture (%)	8.39	9.06
Crude proteins (%)	44.16	44.25
Crude lipids (%)	20.91	21.08
Ash (%)	8.14	11.01
Energy (MJ/kg)	17.95	18.06
Fish meal	110	-
Poultry meal	90	70
Fish trimming meal	-	259.8
Fish hydrolyzed protein	-	15.2
Soybean meal	347	109.9
Hemoglobin	118.3	112
Wheat gluten	22	41.4
Wheat meal	98.7	113.8
Dehulled pea	-	98.4
Fish oil	120	-
Soybean oil	43	32.5
Algal oil	-	88.5
L-Lysine	4	4
DL-Methionine	3	3
Soybean lecithin	5	2.5
Monosodic phosphate	4	4
Mineral–vitamin premix	10	10
Choline	25	35

**Table 2:** Nitrogen water chemistry (mean ± SD) in the tanks of the two diet groups (D1, control diet; D2, experimental diet).

	Day-1	Day-30	Day-60	Day-89
**TAN**				
D1	0.16 ± 0.01	0.35 ± 0.11	0.31 ± 0.02	0.42 ± 0.03 ^a^
D2	0.16 ± 0.01	0.22 ± 0.13	0.31 ± 0.05	0.28 ± 0.01 ^b^
**NO_2_-N**				
D1	0.025 ± 0.01	0.085 ± 0.02	0.086 ± 0.02	0.089 ± 0.01
D2	0.025 ± 0.01	0.085 ± 0.01	0.087 ± 0.01	0.090 ± 0.03
**NO_3_-N**				
D1	0.9 ± 0.2	0.9 ± 0.3	1.18 ± 0.03	1.14 ± 0.06
D2	0.9 ± 0.2	1.0 ± 0.2	1.16 ± 0.02	1.15 ± 0.02

TAN = total ammonia nitrogen (mg/L); NO_2_-N = nitrites (mg/L); NO_3_-N = nitrates (mg/L). ^a,b^ *p* < 0.05 between groups within the same day.

**Table 3:** Growth performance of the fish fed D1 (Control) or D2 (experimental) diet.

Item	D1	D2	*p*-Level
Weight gain	124.0 ± 8.0	116.7 ± 9.0	n.s.
CI	1.4 ± 0.2	1.3 ± 0.3	n.s.
SGR	2.2 ± 0.9	2.1 ± 0.8	n.s.
SR	100.0	100.0	n.s.
FCR	1.03 ± 0.05 ^a^	0.95 ± 0.01 ^b^	<0.05

CI = Condition Index; SGR = Specific Growth Rate; SR = Survival Rate; FCR = Feed Conversion Rate; n.s. = not significant; ^a,b^ *p* < 0.05 between groups.

**Table 4:** Results of the economic analysis performed on the data concerning the diets’ formulation and feeding trial.

Economic Index	D1	D2
CF	0.98	0.83
CFB	1.01	0.79

CF = cost of feed (EUR/kg_feed_); CFB = cost to obtain one kg of fish biomass (EUR/kg_fish_).

## Data Availability

Data are available from the authors upon request.

## Supplementary Materials

The following supporting information can be downloaded at: https://www.mdpi.com/article/10.3390/ani16081188/s1, Table S1: Fatty acid profiles (% of total fatty acids) of the lipid sources used in the trial.

## Abbreviations

The following abbreviations are used in this manuscript:

ANOVA	Analysis of variance
CF	Cost of feed
CFB	Cost of fish biomass
CI	Condition index
DHA	Docosahexaenoic acid
EPA	Eicosapentaenoic acid
FA	Fatty acids
FCR	Feed Conversion Rate
FPH	Fish protein hydrolysates
FTM	Fish trimmings
NO_2_-N	Nitrites
NO_3_-N	Nitrates
PUFA	Poly-unsaturated FA
SGR	Specific Growth Rate
SR	Survival Rate
TAN	Total nitrogen ammonia
