# Supplementary material for: Evaluation of Alternative Protein and Lipid Sources for Rainbow Trout (Oncorhynchus mykiss): Growth, Fillet Quality, and Economic Outcomes of a Farm-Based Diet
Source: Animals (Basel). 2026 Apr 14;16(8):1188. doi: 10.3390/ani16081188 (PMC13113786; doi:10.3390/ani16081188)
Supplement: Supplementary file 1 [file animals-16-01188-s001.zip › animals-4233256-SI.pdf]

Table S1. Fatty acid profile (% of total fatty acids) of the lipid sources used in the trial

	**Soybean oil**	**Fish oil**	**Algal oil**
14:0	0.00	18.24	2.92
15:0	0.00	0.49	0.64
16:0	10.9	16.4	32.4
17:0	0.00	0.98	0.53
18:0	3.90	2.70	2.41
20:0	0.24	0.47	0.62
**Total SFA**	**15.04**	**39.28**	**39.52**
14:1	0.00	0.16	0.00
15:1	0.00	0.00	0.00
16:1	1.50	7.44	0.00
17:1	0.43	0.49	0.00
18:1	21.9	12.5	0.00
20:1	0.00	1.90	0.00
22:1	0.00	0.00	0.36
**Total MUFA**	**23.83**	**22.49**	**0.36**
18:2 n-6	54.32	6.90	0.00
18:3 n-6	0.41	0.00	0.00
20:3 n-6	0.00	0.00	0.10
20:4 n-6	0.00	1.10	2.32
**Total PUFA n-6**	**54.73**	**8.00**	**2.42**
18:3 n-3	5.92	0.85	0.00
18:4 n-3	0.00	2.50	0.20
20:3 n-3	0.00	0.00	0.10
20:4 n-3	0.00	0.00	0.90
20:5 n-3	0.00	18.50	15.10
22:5 n-3	0.00	2.30	1.50
22:6 n-3	0.00	5.20	39.90
**Total PUFA n-3**	**5.92**	**29.35**	**57.70**
n3/n6	0.11	3.67	23.84
